# Coronary Perforation Complicating Percutaneous Coronary Intervention – A Case Illustration and Review

**DOI:** 10.7603/s40602-013-0002-9

**Published:** 2013-10-01

**Authors:** Ang Chin Yong, Jack Tan Wei Chieh

**Affiliations:** Department of Cardiology, National Heart Centre Singapore, Singapore, Singapore

## Abstract

Coronary perforation is a potentially fatal complication during percutaneous coronary intervention (PCI). Reports have shown that it occurs in 0.2 to 0.6% of all patients undergoing the procedures. [1-3] Though the frequency of coronary perforation is low, it is a serious and potentially life-threatening situation that warrants prompt recognition and management. Here we illustrate a case of coronary perforation, and review the incidence, causes, clinical sequelae and management of coronary perforation in the current contemporary practice.

## CASE

**Figure 1:**
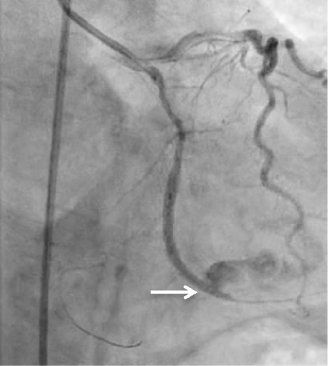
*Type III coronary artery perforation of obtuse marginal branch with contrast extravasation into the pericardium.*

**Figure 2:**
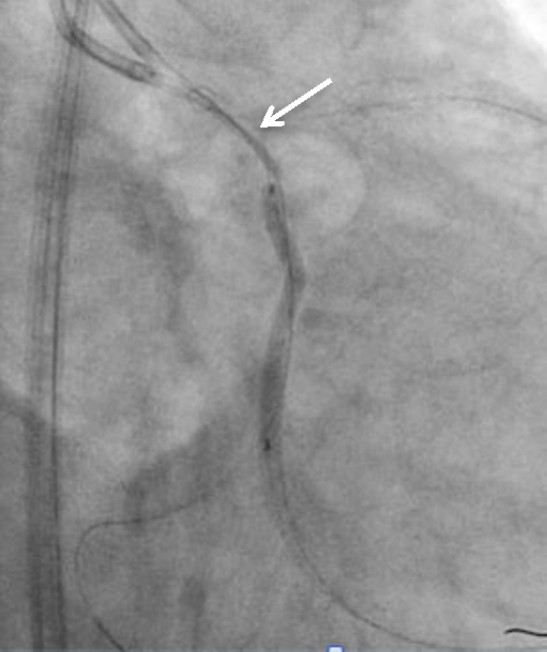
*Dual catheter technique was used during stent delivery. PTFE-covered stent dislodgement (arrow) occurred as a result of angulated and tortuous proximal left circumflex.*

A 61-year old man with known condition of ischemic heart disease was admitted for elective PCI to left circumflex artery chronic total occlusion. PCI attempt to left circumflex chronic total occlusion was carried out. Diagnostic angiography showed diffuse calcified occlusion extending from the proximal to distal left circumflex artery with Renthrop classification grade II collaterals from the diagonals filling up the obtuse marginal branches(OM). With a 6 French extra back-up guider and micro-catheter support, attempts were made initially using soft hydrophilic wire (Asahi Fielder 0.014), followed by intermediate tapered tip wire (Boston Scientific PT2 0.014). Subsequently, by using a stiff wire(Asahi Neo Conquest Pro 0.014), lesion was successfully crossed with wire tip placed at the large OM2. After exchanging to a soft wire and placing a second wire in the distal left circumflex, multiple balloon inflations at various sites from proximal left circumflex to the OM2 using 1.5 x 15mm balloon (10 atmosphere) and 2.0 x 15mm balloon (14 atmosphere) were undertaken. This was followed by further dilation proximally with a non-compliant 2.5 x 15 balloon (20 atmosphere) and placement of a 2.5 x 38mm drug-eluting stent (Abbott Vascular Xience Prime 2.5 X 38), stretching from proximal left circumflex to the OM2 branch (14 atmospheric pressure). Angiography post stent deployment showed an Ellis type III coronary artery perforation at the OM2. (Figure 1) Prolonged balloon inflation proximal to the site of perforation failed to seal the perforation. Using dual catheter technique, a second guider(7F) is inserted via left femoral arterial while prolonged balloon inflation was performed through the initial guiding catheter. A second guidewire is then advanced through the second guider, and into the affected OM branch upon balloon deflation for delivery of the covered stent. Attempt to deliver a premounted PTFE covered stent (InSitu Direct- Stent Stent Graft 2.5 x 19mm) however, was unsuccessful as the stent was dislodged due to tortuous proximal left circumflex segment. The stent was later captured and removed by using a microsnare whilst balloon was inflated in the circumflex artery. (Figure 2) Patient began to become hemodynamically unstable with large pericardial effusion visualized on fluoroscopy. Pericardiocentesis was performed and repeat angiography showed persistent type III perforation. The patient was taken for emergency coronary artery repair and CABG. The site of perforation was sought and a 8mm tear at the OM2 was identified. The patient recovered post-surgery with no deficits and was subsequently discharged 10 days later.

## DISCUSSION

### Incidence, Classification and Clinical Outcome

Coronary perforation during percutaneous coronary intervention is a well-recognized but undesirable complication due to its lifethreatening consequences e.g. cardiac tamponade, acute myocardial infarction. (4,5) It occurs when an anatomical breach in the integrity of coronary vessel wall is present as a result of penetration, intimal tear, or dissection that propagates outward, leading to extravasation of blood, either into the myocardium, pericardium or a cardiac chamber. Studies have reported that it occurs in 0.2 to 0.6% of all patients undergoing coronary intervention procedures. [1,2,3] The incidence of perforation, whilst low, is increased in complex interventional procedures and with the use of debulking devices.[6,7] (Table 1)

**Table 1:** *Incidence of Coronary Perforations with Usage of Debulking Devices.* [6]

Device	Incidence
Incidence Balloon angioplasty only	0.1%
Directional atherectomy	0.7%
Excimer laser	1.9%
Rotational atherectomy	1.3%
Transluminal extraction catheter	2.1%

* Modified from Ellis SG et al. Increased coronary perforation in the new device era. Incidence, classification, management, and outcome. Circulation 1994; 90: 2725-30

The clinical risk after perforation can be classified angiographically, but even low-risk perforations occasionally have poor clinical outcome. Several classification methods for coronary artery perforation have been published (Table 2).[6,8,9] Ellis classification scheme is the most widely used classification for risk stratification and prognostication.[6] Ellis et al evaluated a multicenter registry of 12,900 PCIs, in which 62 perforations were reported and classified angiographically into 3 types. Type I CAP is defined by the development of an extraluminal crater without extravasation. Type II CAP is defined by the development of a pericardial or myocardial blush without contrast jet extravasation. Type III CAP is defined by the development of an extravasation jet through a frank (≥1 mm) perforation or cavity spilling into an anatomic cavity chamber (ventricles, pericardial space, etc). Several studies further divide Ellis type III into type III and type IV (without and with cavity spilling, respectively) as patients with type III cavity spilling typically fare better in terms of outcomes. [10,11]

**Table 2:** *Classification of Coronary Artery Perforation.* [6, 8, 9, 41]

		Clinical Outcome (%)		
Classification	Description	Tamponade	Emergent CABG	Death
Ellis[6,41]	Type I: Extraluminal crater without extravasation	6-8	15-24	0-6
	Type II: Pericardial or myocardial blush without contrast jet extravasation	5-13	10-24	0-6
	Type III: Extravasation jet through a frank(≥1mm) perforation towards pericardium	20-63	50-60	19-21
	Type IV (previously known as Type III cavity spilling): perforation into an anatomic cavity chamber, coronary sinus, etc	0	0	0
Fukutomi[8]	Type I: Epicardial staining wihout contrast extravasation			
	Type II: Epicardial staining with a visible jet of contrast extravasation			
Kini[9]	Type I: Myocardial staining without contrast extravasation			
	Type II: Constrast extravasation into pericardium, coronary sinus or cardiac chambers			

Clinical outcome relates to the severity of the coronary perforation as graded by the Ellis classification. [Table 2] From analysis of a Canadian pooled data, patients with Ellis type II perforation had higher rates of tamponade and myocardial infarction compared with patients with Ellis type I perforation; however, mortality rates were similar between the 2 groups. Ellis type III patients had the highest rate of adverse events, with a more than 10- fold increase in tamponade and death compared with Ellis type II patients.[12] Another Italian series also showed high inhospital mortality at 14.8% and myocardial infarction (42.9%) in patients with large type III perforation. [13]

### Risk Factors and Causes

Coronary perforation can be caused by vessel penetration from inadvertent guidewire exit, vessel tear due to oversized balloons, stents or balloon rupture, or anatomical breach in vessel wall from excessive de-bulking during PCI. High-risk angiographic predictors included type B or C lesions, chronic total occlusion, calcified disease, small, angulated or tortuous vessels, and the presence of multivessel coronary disease. [7,10,12] Other clinical predictors, which are likely interrelated are elderly patients, female and PCI for acute coronary syndrome. [6]

Perforation during PCI is most likely to occur when advancing the guidewire, or when the balloon is dilated, or when balloon ruptures. Just as dissection frequently occurs during balloon angioplasty, over-dilatation or in the event of balloon rupture can cause a dissection all the way into the adventitia which leads to perforation. This event typically occurs when the balloon to artery ratio exceeds 1.1 to 1.3. [6,14,18]. A balloon-to-artery ratio >1.1 was reported by Ajluni SC et al to be associated with a 2 to 3-fold increase in perforation. [18] Guidewire perforation is especially prevalent with the use of stiff hydrophilic wires when treating CTOs. [15,16,17] Javaid et al. and Kiernan et al. reported that more than 85% of coronary perforations occurred with the use of hydrophilic guidewires. [16,17]

The development of debulking devices such as in excimer laser angioplasty, directional and rotational coronary atherectomy may be effective for obtaining larger lumen areas, but they are also associated with significant higher rates of coronary perforation. [6,15] Ellis et al. reported greater incidence of perforation (2.1%) with devices that removed rather than displaced tissue [6] while recent registry data including patients from 2 European centres by Rasha Al-Lameee at al. reported incidence rate of 3.6% with rotablators and directional atherectomy [13] Treatment of heavily calcified or resistant stenosis however involves these methods, and a high incidence of severe dissections and perforations ensues.

Concomitant IIb/IIIa antagonist use increases the complication rate and diminishes the ability to seal a perforation successfully. [19] Stankovic et al. found a trend for a higher rate of coronary perforation with the use of IIb/IIIa inhibitors (OR, 1.86),[7] meanwhile Fasseas et al. found that 33.3% of those receiving IIb/IIIa inhibitors required placement of covered stent or emergency cardiac surgery, compared with 3.2% of patients who did not receive IIb/IIIa inhibitors. [10] Subgroup analysis of data by Al-Lamee R, at al showed higher proportion of GP IIa/IIIa patients who needed multiple treatment methods for coronary perforation. In addition, the procedural and in-hospital MACE rate was also higher (90.0%). [13]

In this PCI case involving chronic total occlusion, though lesion was successfully crossed, subsequent oversized balloon inflation and stenting led to tear in vessel wall and grade III perforation. Always bear in mind, that irrespective of what device you are using, the danger of perforation is increased in complex cases such as CTOs, bifurcations, tortuous lesions, and those on proximal bends and calcified lesions.

### Management of Coronary Perforation

The risk of perforation exists the moment the guidewire is inserted, and remains throughout PCI procedure. Preventive measures to avoid coronary artery perforation include meticulous attention to guidewire position, careful and appropriate sizing of the balloon or stent prior to inflation, and avoiding overdilation or high pressure inflation exceeding the balloon’s burst pressure. Extra care must be taken in high-risk lesion types, e.g. calcified, angulated, bifurcation lesions or chronic total occlusion, as well as during the usage of debulking device. This shall include gentle handling of equipment and frequent angiographic shots to detect perforation promptly should it occur.

**Figure 3:**
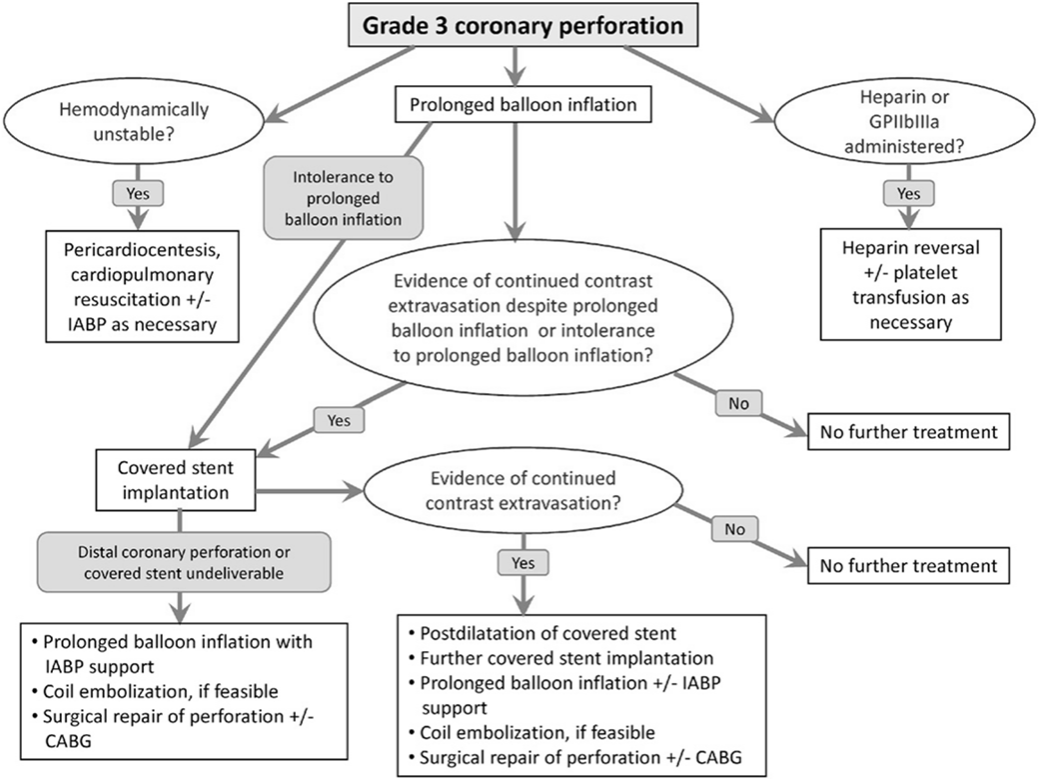
*Algorithm for the Management of Coronary Artery Perforation.*

The management of coronary perforation remains challenging, involving continuous assessment of the hemodynamic status while carrying out attempt to seal the perforation. Once coronary artery perforation is identified, prompt management is crucial to avoid emergent surgery and devastating sequelae. (Figure 3) Blood flow at the perforated branch can be blocked by prolonged balloon inflation. This can often be promptly carried out at low pressure (1-2 atmosphere) and should last for 5 to 30 minutes to promote hemostasis at the perforated site and may avoid surgery in two third of cases.[20] If myocardial ischemia develops, the plain balloon should be exchanged with the perfusion balloon that enables perfusion to the distal vessel.

*An algorithm for the management of grade III coronary perforation suggested by Al Lamee, et al.*[13] *(CABG - coronary artery bypass graft; GPIIbIIIa - glycoprotein IIb/IIIa inhibitors; IABP - intraaortic balloon pump)*

There are conflicting opinions on the benefits of reversing anticoagulation with protamine sulphate and the risks of inducing de novo or stent thrombosis. Systemic protamine to reverse the effect of heparin should be considered in the event of continuous extravasation with hemodynamic compromise despite initial effort to achieve hemostasis. Dosage administered (1mg per 100 units unfractionated heparin administered) should aim to reduce activated clotting time (ACT) to less that 150 seconds or partial thromboplastin time to less than 60 seconds.[17,21,24] The introduction of newer antithrombotic agent such as bivalirudin and GP IIb/IIIa inhibitors requires discontinuation of such therapy. Platelet transfusion can be administered to reverse the effect of abciximab, but not for tirofiban or eptifibatide.[22]

Patient’s hemodynamic status must be monitored closely, administration of intravenous fluids could prevent cardiac tamponade and hypotension. Portable transthoracic echocardiography is a useful to assess pericardial effusion and tamponade. Development of cardiac tamponade is associated with poor outcomes with high morbidity and mortality with reported emergent coronary bypass graft surgery rate of 33% and death rate of 25%.[23] Percardiocentesis should be performed immediately eventhough aspiration and placement of a pericardial drain in an emergency can be technically challenging.[8]

**Figure 4[28,39]:**
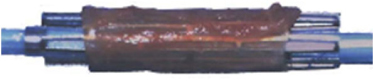

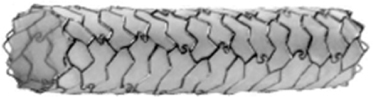
*a. Autologous vein graft covered stent* *b. PTFE-covered stent (JOSTENT Graftmaster)*

If coronary artery perforation persists despite prolonged balloon inflation and reversal of anticoagulation, a covered stent can be used, especially for perforation at the proximal or mid-segment of the involved artery. Autologous vein graft stent has been used successfully before the commercially available covered stents with poly-tetrafluorethylene( PTFE) and pericardium. [36,37,38,39] This stent needs to be assembled by the operator during the procedure; thus takes time and prolonged balloon inflation at the perforation site is required.(Figure 4) A saphenous, antecubital, or cephalic autologous vein is harvested surgically and fixed to a bare metal stent with the stent then be hand-crimped onto a balloon. Successful closure of a coronary perforation with a make-shift stent sandwich by cutting a cylindrical portion of balloon material and used it as a membrane sand-wiched between two stents, was also reported.[40]

PTFE-covered stents have been commercially available and widely used for coronary perforation. Reports have shown that PTFEcovered stents are effective way to achieve hemostatsis without resorting to emergency CABG.[18,25,26,27,28](Figure 4) From a multinational retrospective registry by Lansky AJ, et al, PTFE-covered stent appeared highly effective with 92.9% success rate in achieving complete seal of grades 2 and 3 perforation.[28] However, it is important to recognize that covered stents are bulky and lack of flexibility. Rapid stent delivery and proper positioning in tortuous and calcified vessel can be technically difficult or impossible, as illustrated in the case presented above where stent dislodgement occured.(Figure 2) Dual catheter technique described by Ben-Gal Y, et al is useful to allow delivery of the covered stent without losing control of the perforation site. In this technique, a second guider is inserted via a new arterial access while prolonged balloon inflation was performed through the initial guiding catheter. A second guidewire is then carefully advanced through the second guider, into the affected vessel distal to perforation upon balloon deflation for delivery of the covered stent.[29] This technique is particularly helpful in large perforation which requires ongoing sealing of the perforation while delivering the covered stent, as well as providing better support for the stent delivery.

Another important concern of PTFE-covered stents is the high rate of stent thrombosis and restenosis. Al-Lamme, et al reported 8.6% incidence of definitive stent thrombosis in patients treated for coronary perforation; all of these cases were associated with covered PTFE stent implantation, suggesting the higher thrombogenicity of these stents, in combination with the increased risk of ST conferred by coronary perforation.[18] Another study on patients treated with PTFE covered stent suggested a decrease in stent thrombosis with the use of IVUS-guided PCI, postdilatation, and prolonged thienopyridines therapy (3–6 months).[30] In longer term, covered stents have also been associated with high rates of in-stent restenosis and therefore repeated procedures of revascularization (reported up to 50%).[31,32]

For distal artery perforation which the vessel caliber is small, embolization with polyvinyl alcohol, collagen foam, intracoronary thrombin, or thrombogenic metallic coils into the leaking vessels can be a treatment option. This should be considered when the perforation is too distal or in a small vessel where stent implant or surgery is not possible.[33,34,35]

It is important to realize the limitations of nonsurgical devices if the damage to the vessel is substantial. If the above methods fail to seal a perforation that is causing serious ischemia, or covered stent delivery is unsuccessful as illustrated in the case above, emergency surgery with perforation repair and bypass grafts should be considered.[4,5] A perfusion balloon can be placed to temporarily contain the perforation while preparing the patient for emergent surgery.

### Conclusion

While performing PCI, it is important to recognize patients at high risk for coronary perforation and caution must be taken to prevent this dreadful complication. Early detection and prompt management is crucial to avoid devastating sequelae. Management involves not only effort to contain the perforation, patient’s hemodynamic status must also be assessed continuously and pericardiocentesis should be performed if cardiac tamponade develops. While prompt surgical intervention may be life saving, expertise in the use of covered stents, and gel foam or coil embolization in selected cases can frequently provide a valuable rescue option without resorting to emergent surgery. Caution should be exercised and limitation of these non-surgical devices should be recognized while employing these treatments.
